# Picosecond Pulsed Laser Deposition of Metals and Metal Oxides

**DOI:** 10.3390/ma16196364

**Published:** 2023-09-22

**Authors:** Anna Dikovska, Genoveva Atanasova, Tina Dilova, Aleksandra Baeva, Georgi Avdeev, Petar Atanasov, Nikolay Nedyalkov

**Affiliations:** 1 Institute of Electronics, Bulgarian Academy of Sciences, 72 Tsarigradsko Chaussee, 1784 Sofia, Bulgaria; dikovska@ie.bas.bg (A.D.); paatanas@ie.bas.bg (P.A.); nned@ie.bas.bg (N.N.); 2 Institute of General and Inorganic Chemistry, Bulgarian Academy of Sciences, Acad. G. Bonchev Str., bl. 11, 1113 Sofia, Bulgaria; genoveva@svr.igic.bas.bg (G.A.); alexandravbaeva@gmail.com (A.B.); 3 Rostislaw Kaischew Institute of Physical Chemistry, Bulgarian Academy of Sciences, Acad. G. Bonchev Str., bl. 11, 1113 Sofia, Bulgaria; g_avdeev@ipc.bas.bg

**Keywords:** PLD, picosecond ablation, metals, metal oxides

## Abstract

In this work, we present the fabrication of thin films/nanostructures of metals and metal oxides using picosecond laser ablation. Two sets of experiments were performed: the depositions were carried out in vacuum and in air at atmospheric pressure. The subjects of investigation were the noble metals Au and Pt and the metal oxides ZnO and TiO_2_. We studied and compared the phase composition, microstructure, morphology, and physicochemical state of the as-deposited samples’ surfaces in vacuum and in air. It was found that picosecond laser ablation performed in vacuum led to the fabrication of thin films with embedded and differently sized nanoparticles. The implementation of the same process in air at atmospheric pressure resulted in the fabrication of porous nanostructures composed of nanoparticles. The ablation of pure Pt metal in air led to the production of nanoparticles with an oxide shell. In addition, more defects were formed on the metal oxide surface when the samples were deposited in vacuum. Furthermore, the laser ablation process of pure Au metal in a picosecond regime in vacuum and in air was theoretically investigated using molecular dynamics simulation.

## 1. Introduction

Recently, femtosecond (*fs*) lasers have become increasingly common and have been intensively studied with respect to their uses in various scientific and industrial fields [1,2]. In particular, applications of *fs* lasers in micro-machining, producing nanoparticles, and forming nanostructures with advanced applications have quickly developed, which have been accompanied by numerous in-depth studies in the field of light–matter interactions [3,4,5]. However, due to their high price and significant service and maintenance costs on the one hand and the unique working environment requirements on the other, *fs* lasers have currently found limited industrial applicability. At the same time, the particular class of picosecond (*ps*) laser systems that emit pulses with a duration of up to several tens of picoseconds have generated considerable interest for scientists and technologists. The interaction of such pulses with matter retains the key traits of pulses conducted with *fs* laser pulses: a strong thermal effect limitation, lack of a large heat-affected area around the impact area, and realization of non-thermal mechanisms of phase transformations, such as phase explosion and homogeneous melting. Being less complicated and more reliable, these laser systems are considerably less expensive than *fs* laser systems.

Many of the works involving *ps* laser systems that have been reported so far have been stimulated by possibilities for applications, such as cutting, drilling, melting, etc. [6,7,8,9]. Thus, they have been primarily focused on modifying the target’s morphology with no particular interest in the detailed description of the properties and dynamics of the species that are evaporated and ablated. Furthermore, several studies have reported the use of *ps* lasers for the fabrication of thin films and/or structures and have discussed their possible applications [10]. Huotari et al. described the picosecond laser deposition of a metal oxide nanostructure for high-sensitivity gas sensor applications [11]. Additionally, Kekkonen et al. reported the picosecond pulsed laser deposition of metal-oxide sensing layers with controllable porosity, again with respect to gas sensor applications [12]. Some authors investigated the effects of the picosecond deposition of metal oxides at high pulse repetition rates [13,14]. However, all of these studies have been focused on the properties of the produced films/structures rather than on the mechanism of their fabrication and the dynamics of the ablated species. On the contrary, some authors have only been focused on the theoretical investigation of the mechanisms of ultrashort laser–matter interaction without further interest in the ablated material [15,16].

The aim of this work was to study the material removed from the target when performing picosecond laser ablation. The laser ablation process is the basis of the technology that is used for thin film/nanostructure fabrication, namely, pulsed laser deposition (PLD). Applying *ps* pulses for PLD preserves the features of an ultrashort laser–matter interaction and advances this technology toward its commercial use. This defines the interest in *ps*-PLD from a scientific and research perspective. The subject of investigations in this study were the noble metals Au and Pt and the metal oxides ZnO and TiO_2_. The nanoparticles and nanostructures of noble metals such as Au and Pt are of great scientific interest due to the presence of efficient plasmon excitations with resonances in the near UV and visible spectral ranges [17]. A variety of novel applications in nanoelectronics, optical imaging, biomedicine, telecommunications, photovoltaics, photocatalysis, etc., all revolve around field localization and enhancement. ZnO is an important technological material for the effective design of optoelectronic devices, ultraviolet light emitters, photovoltaics, transparent and spin electronics devices, biosensors, etc., due to its useful mechanical, electrical, and optical properties as well as its relatively easy fabrication and low production costs [18]. Among oxides, TiO_2_ is a promising semiconductor material because of its high chemical stability, high refractive index, surface functionality, self-cleaning and photocatalytic activity/recycling, antibacterial properties, and biocompatibility [19]. Furthermore, PLD is a flexible technology that allows for the easy fabrication of composite materials, which considerably enriches the possible application of the relevant metal oxides and noble metals.

We studied and compared the phase composition, microstructure, morphology, and physicochemical state of the surface of samples deposited by *ps*-PLD in vacuum and in air. Furthermore, a theoretical investigation of the laser ablation process of Au metal in a picosecond regime in vacuum and in air was implemented using molecular dynamics simulation in order to clarify the mechanism of material decomposition during *ps* pulse irradiation.

## 2. Materials and Methods

### 2.1. Experimental

The samples were produced by applying picosecond pulsed laser deposition (PLD). The ablation process was carried out using a picosecond Nd:YAG laser (CNI Laser, PS-A1-1064) operating at a fundamental wavelength of 1064 nm with a pulse duration of 10 ps and a repetition rate of 1 kHz. The laser fluence applied on the target was 0.45 J/cm^2^. The targets used for ablation were the noble metals Au and Pt and the metal oxides ZnO and TiO_2_. We used quartz and silicon as substrates. Two sets of experiments were performed: the depositions were carried out in vacuum and in air at atmospheric pressure. The experiments in vacuum were conducted at a target–substrate distance of 30 mm and a base pressure of 10^−4^ Torr. In air, the target–substrate distance was kept at 5 mm. All depositions were performed at room substrate temperature for 3 min.

### 2.2. Theoretical

In order to study the dynamics of a metal target interacting with a *ps* laser pulse, a molecular dynamic (MD) model was developed. It is based on the classical MD approach, whereby the time evolution of an ensemble of atoms can be described by a set of equations of motion for each atom [20]:

(1) $$m_{i}\frac{\partial^{2}r_{i}}{\partial t^{2}}=-\nabla_{i}U\left(r_{1},r_{2},\ldots \ldots ,r_{N}\right)$$

where *m_i_* and *r_i_* are the mass and coordinate of the atom, respectively, and *U* (*r*_1_, *r*_2_, *…*, *r_N_*) is the interatomic potential. One of the main advantages of the MD method is that the initial input parameters are only the atoms’ positions, the temperature of the system (velocities), and the potential of interaction. No additional consideration and approximations are necessary to obtain the time evolution of the simulated system. In the case presented in the simulation of Au, the simulated system was constructed by 150 × 1 × 200 cubic face-centered unit cells. The initial temperature was 300 K. The interaction between the atoms of the system was considered to be adequately described by the Morse potential, and the specific parameters for Au are taken from [21]. The sets of equations in Equation (1) is solved by the “velocity Verlet” algorithm [20]. Along the lateral direction of the simulation system, periodic boundary conditions are imposed, thus simulating the central part of the laser spot. When the laser pulse duration is shorter than or is in the order of the specific time for energy transfer between the electron system that initially absorbs the laser radiation, the lattice temperature evolution of the system should be described by a two-temperature diffusion model, which consists of one part for the electron system and another part for the lattice [22]. This approach was used in the MD model in order to simulate the interaction with the *ps* laser pulse. The heat diffusion equation was used to predict the temperature of the system, which was set in the atomic system of MD using an appropriate scaling of the atomic velocities. Further details on the simulation model and parameters used can be found in [23].

The MD model was also used to estimate the role of the presence of a high-pressure gas phase in the ablation material dynamics. This case corresponds to ablation in air at atmospheric pressure. Because the real interactions of the ablated species with air, with all of its components, are complex and difficult to describe using an MD model, a simplified interaction is assumed. The main effect of the presence of gas at high pressure on the ablation plume is confinement of the plume close to the target surface [24,25] by decreasing the ablated species’ velocities. In the model presented, this effect is accounted for by scaling the ablated atoms’ velocities using an appropriate parameter, whose value is estimated based on the data available on the plume velocity distribution in laser ablation in air [26].

### 2.3. Sample Characterization

Scanning electron microscopy (SEM) was conducted using a LYRA I XMU system (Tescan, Brno, Czech Republic) to study the samples’ morphology. Transmission electron microscopy (TEM) and selected area electron diffraction (SAED) images were taken by a JEOL JEM 2100 system (Akishima-Shi, Tokyo) to reveal the samples’ microstructure and crystallinity. Some of the samples for TEM analyses were prepared by scratching the sample surface in a drop of distilled water and transferring a small quantity of the material to a Cu grid. Samples for TEM analyses were also prepared using a direct deposition on the TEM Cu grid in the case of ablation of a Au target in order to compare the obtained experimental results with the theoretical ones. In this case, the depositions were performed for a shorter deposition time (1 min) in order to prevent nanoparticles overlapping. X-ray diffraction (XRD) using an Empyrean diffractometer (PANalytical, Malvern, UK) was used to examine the samples’ crystalline structure and phase composition. The chemical composition of the samples’ surface was examined using X-ray photoelectron spectroscopy (XPS) on an AXIS Supra electron spectrometer (Kratos Analytical Ltd., Manchester, UK).

## 3. Results

### 3.1. Experimental Part

#### 3.1.1. Noble Metals—Au and Pt

**Figure 1:**
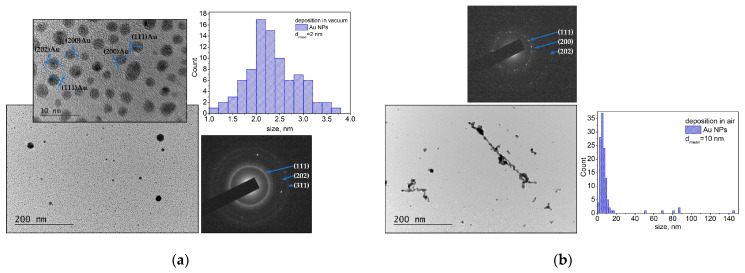
TEM image, size distribution, and SAED pattern of material ablated from the Au target (**a**) in vacuum and (**b**) in air. The samples were prepared by direct deposition on the TEM Cu grid.

Figure 1 illustrates the microstructure of the samples obtained by the laser ablation of the Au target. The samples were prepared through a direct deposition on the TEM Cu grid. The TEM image and size distribution of the material deposited in vacuum are presented in Figure 1a. As can be seen, the material ablated from the Au target in vacuum mainly consists of spherically shaped small nanoparticles with sizes in the range of 1–4 nm and a mean diameter of 2 nm. However, larger particles with a size of 40–80 nm are also observed. The SAED image demonstrates that the nanoparticles are crystalline (Figure 1a) and the interplanar distance from the main reflections can be assigned to a family of planes of metallic Au (gold, cubic, ICPDS 96-110-0139).

The TEM image and size distribution of the material deposited in air are presented in Figure 1b. The ablated material consists of individual nanometer-scale particles and nanoaggregates with different sizes and shapes. The corresponding size distribution shows that most of the nanoparticles have diameters ranging from 1 nm to 20 nm, with the mean diameter being 10 nm. All nanoparticles and nano-agglomerates are crystalline, which is confirmed by the SAED pattern (Figure 1b).

**Figure 2:**
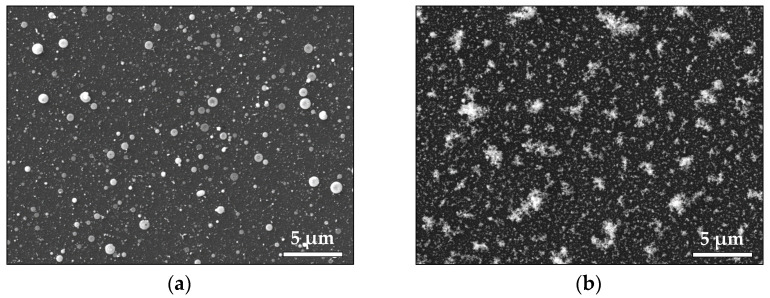
SEM image of the Au sample produced (**a**) in vacuum and (**b**) in air.

SEM images of the Au samples produced in vacuum and in air are shown in Figure 2. The sample deposited in vacuum (Figure 2a) displays a flat morphology with a significant amount of droplets, with sizes ranging from 80 nm to 600 nm. An SEM image of the Au sample deposited in air is shown in Figure 2b. The deposition process in air leads to the formation of a fine structure consisting of an ensemble of smaller particles. The XPS analyses of the Au samples that were performed to reveal the physicochemical state of the samples (Figure S1) found no difference in the XPS spectra of the Au samples prepared in vacuum and in air. The presence of Au was confirmed by registering the characteristic Au 4f peak at a binding energy of 84.0 eV, which corresponds to bulk metallic gold (Figure S1).

**Figure 3:**
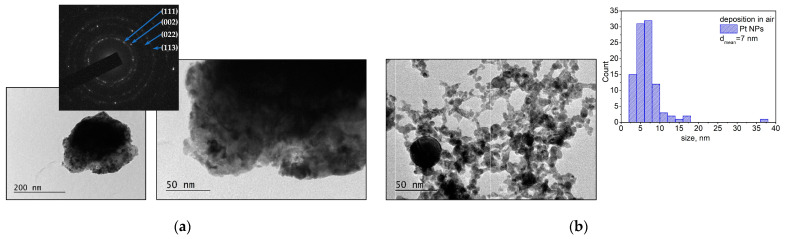
(**a**) TEM image, corresponding size distribution, and SAED patterns of material ablated from the Pt target in vacuum; (**b**) TEM image and size distribution of material ablated from the Pt target in air. The samples were prepared by transferring a small quantity of the material to a TEM grid.

TEM images of the samples produced by ablation from a Pt target in vacuum and in air are shown in Figure 3. The samples for TEM analyses were prepared by transferring a small quantity of the material that was deposited on a substrate to a TEM grid, as described in the Section 2.3. Figure 3a shows the TEM images and a SAED pattern of the material ablated from the Pt target in vacuum. In the structure formed on the substrate, nanoparticles with sizes in the range of 2–10 nm can be recognized. The SAED pattern indicates that the structure is crystalline, with the patterns being indexed to metallic Pt (platinum, cubic, ICPDS 96-101-1112).

A TEM image and the size distribution of the material deposited in air are presented in Figure 3b. The nanoparticles and aggregates produced by the ablation of the Pt target in air form a chain-like nanostructure on the substrate. The corresponding SAED pattern shows that the nanostructure is crystalline, and the main reflections can be assigned to a family of planes of metallic Pt.

**Figure 4:**
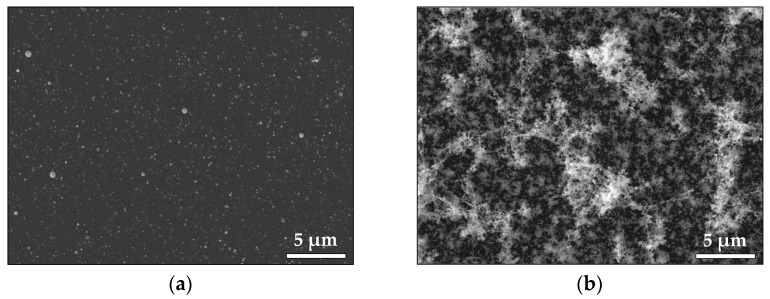
SEM image of the Pt sample fabricated (**a**) in vacuum and (**b**) in air.

Figure 4 illustrates the morphology of the Pt samples produced in vacuum and in air. We observe that the Pt sample deposited in vacuum (Figure 4a) has a flat morphology with many droplet formations with sizes in the range of 80–200 nm, while the same deposition performed in air produces a morphology consisting of distinct features, where it is formed by nano-sized particles (Figure 4b).

**Figure 5:**
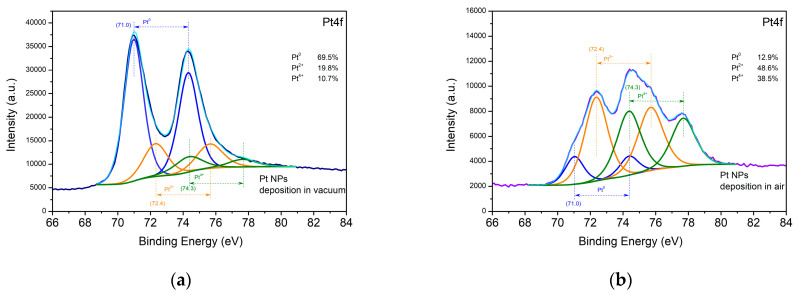
XPS spectra of the Pt samples deposited (**a**) in vacuum and (**b**) in air.

Figure 5 displays high-resolution XPS spectra of Pt 4f taken from the samples deposited from the Pt targets in vacuum and in air. The Pt 4f spectra of both samples were fitted to three major components attributed to metallic Pt^0^ at 71.0 eV and oxidized Pt in different oxide states, respectively, Pt^2+^ at 72.4 eV, and Pt^4+^ at 74.3 eV [27]. Metallic Pt^0^ is deposited on the surface of the sample in a significantly larger amount in vacuum than in air.

#### 3.1.2. Metal Oxides—ZnO and TiO_2_

**Figure 6:**
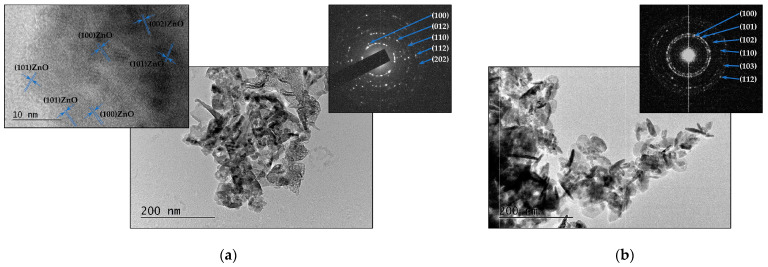
(**a**) TEM image, corresponding SAED pattern, and HR TEM image of material ablated from ZnO target in vacuum; (**b**) TEM image and SAED pattern of material ablated from ZnO target in air. The samples were prepared by transferring a small quantity of the material to a TEM grid.

The TEM images of the samples produced by ablating a ZnO target are shown in Figure 6. Figure 6a displays the TEM image, corresponding SAED pattern, and high-resolution (HR) TEM of the material deposited on the substrate when the experiments were performed in vacuum. The microstructure of the sample is represented by a polycrystalline structure with embedded nanoparticles with irregular form. The HR TEM image reveals lattice fringe spacings of 2.81 Å, 2.60 Å, and 2.48 Å, which correspond well to the (100), (002), and (101) planes of the ZnO hexagonal wurtzite structure (zinc oxide, hexagonal, ICPDS 96-901-1663). The microstructure of the ZnO sample prepared in air consists of separate nanosized clusters of nanoparticles with irregular shapes and sizes.

Elongated nanoparticles/nanorods with lengths ranging from 25 nm to 65 nm and diameters ranging from 7 to 15 nm are clearly visible in Figure 6b. The SAED pattern demonstrates the crystal nature of the structure (Figure 6b). The phase composition and crystal structure of the sample were also confirmed using the XRD analysis (Figure S2).

**Figure 7:**
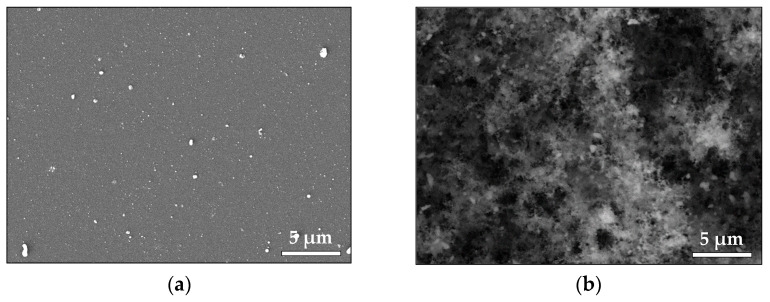
SEM image of material ablated from ZnO target (**a**) in vacuum and (**b**) in air.

The SEM images in Figure 7 illustrate the morphology of the deposited ZnO samples. Figure 7a shows the morphology of a thin ZnO film deposited in vacuum. We observe that the surface is littered by mostly spherical particles/droplets with sizes ranging between 60 and 500 nm. Droplets with irregular shapes are also observed. Figure 7b presents the morphology of the ZnO sample deposited in air. A highly porous structure consisting of aggregated nanoparticles is observed. XPS analyses of the ZnO samples’ surface were also performed. No difference was observed in the Zn 2p XPS spectra of the surface of the samples deposited in vacuum and in air.

We conclude that the Zn atoms are in the Zn^2+^ oxidation state based on the binding energy positions, the width of the Zn 2p peaks, and the spin–orbit splitting of 23.1 eV (Figure S3) [28]. The asymmetric O 1s peak indicates the presence of different oxygen-containing species (Figure S3); it could be fitted by three components that are assigned to lattice O^2−^ ions in the stoichiometric environment of Zn^2+^ ions, oxygen vacancies formed in oxygen-deficient regions of the ZnO lattice, and the presence of adsorbed hydroxyl, carbonate, or O_2_ species, respectively [29]. It should be noted that the components associated with the oxygen deficiency state and with the presence of adsorbed species are amplified in the case of deposition in vacuum.

**Figure 8:**
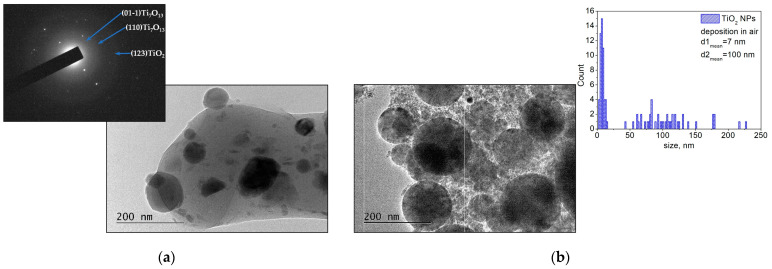
(**a**) TEM image and corresponding SAED pattern of material ablated from the TiO_2_ target in vacuum; (**b**) TEM image and size distribution of material ablated from the TiO_2_ target in air. The samples were prepared by transferring a small quantity of the material to a TEM grid.

In Figure 8, TEM images are presented of the samples fabricated by ablation of the TiO_2_ target in vacuum and in air. As seen in Figure 8a, the morphology of the sample deposited in vacuum has a polycrystalline structure with embedded nanoparticles with sizes ranging from 20 nm to 110 nm and a predominantly spherical shape. The main reflection of the SAED pattern can be identified as non-stoichiometric (Ti_7_O_13_, triclinic, ICPDS 96-100-8197) and anatase (TiO_2_, tetragonal, ICPDS 96-101-0943) phases of titanium oxide. Figure 8b shows a TEM image of the sample deposited by ablation of the TiO_2_ target in air. The microstructure of the sample consists of spherically shaped big particles with sizes mainly ranging from 60 nm to 130 nm (a mean diameter of approximately 100 nm). Furthermore, nanoparticles with sizes in the range of 2–20 nm (a mean diameter of approximately 7 nm) are clearly distinguished (size distribution in Figure 8b).

**Figure 9:**
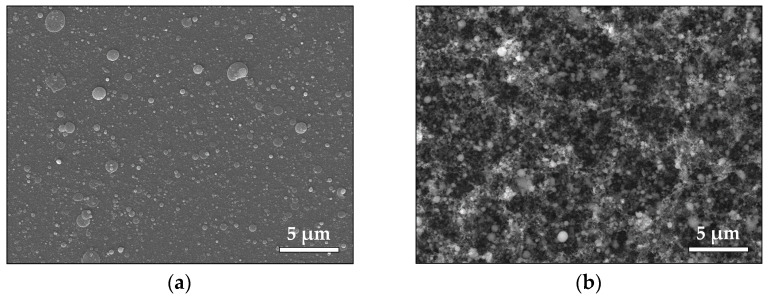
SEM image of material ablated from the TiO_2_ target (**a**) in vacuum and (**b**) in air.

The phase composition of the sample identified by XRD analysis (Figure S4) shows that the sample structure is a combination of anatase and rutile phases of TiO_2_ (rutile, tetragonal, ICPDS 00-021-1276). SEM images of the TiO_2_ samples deposited in vacuum and in air are shown in Figure 9. Figure 9a demonstrates the morphology of the sample deposited in vacuum. One can see that the sample surface is covered by mostly spherical particles that range in size from 100 nm to 1 μm. The morphology of the TiO_2_ sample deposited in air (Figure 9b) is a porous structure that is composed of nanoparticles of different sizes. However, separate droplet formations with sizes ranging 80–700 nm are observed across the entire structure.

**Figure 10:**
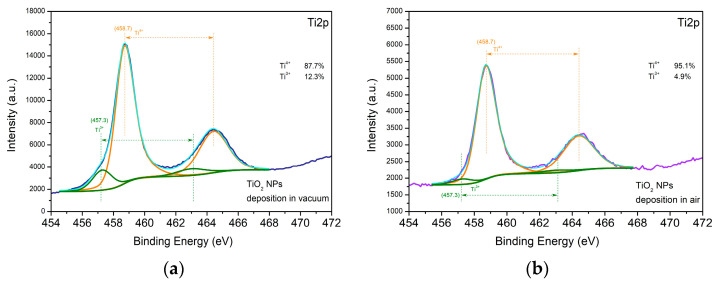
XPS spectra of the TiO_2_ samples deposited (**a**) in vacuum and (**b**) in air.

The high-resolution XPS spectra of the Ti 2p core level of the TiO_2_ sample surface are shown in Figure 10. The Ti 2p spectra can be deconvoluted into two close components at 458.7 and 457.3 eV. The Ti 2p_3/2_ and Ti 2p_1/2_ peaks located at 458.7 eV and 464.0 eV with a separation between the binding energies of 5.7 eV correspond to Ti^4+^ [30]. The Ti 2p_3/2_ and Ti 2p_1/2_ peaks located at 457.3 eV and 464.0 eV are associated with the presence of Ti^3+^ [30]. The amount of the Ti^3+^ component on the surface of the sample deposited in vacuum is more than twice as high as that on the surface of the sample produced in air (Figure 10a,b). As indicated by the asymmetric O1s peak, different oxygen-containing species are present. The peak can be deconvoluted into components that are attributed to lattice O^2−^ ions in the metal oxides as well as to oxygen vacancies and the presence of species adsorbed on the sample surface. The percentage of the components associated with the oxygen deficiency state and with the presence of adsorbed species is higher in the case of deposition in vacuum.

### 3.2. Theoretical Part

**Figure 11:**
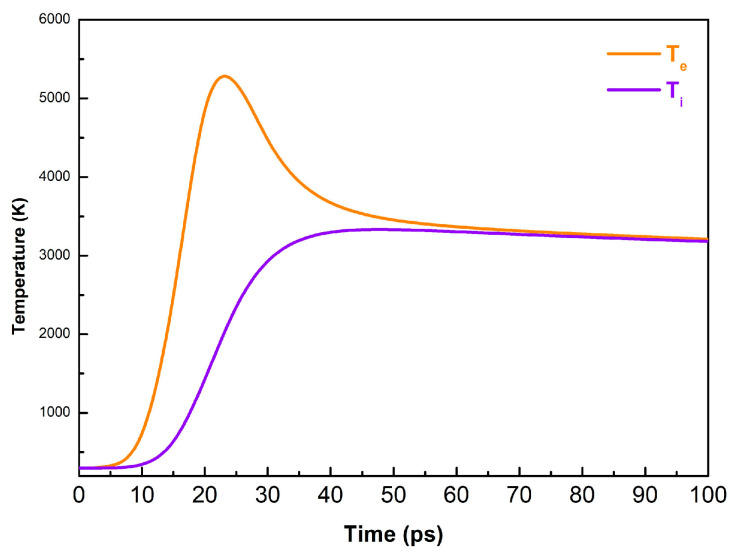
Calculated time evolution of the electron and lattice temperature of Au irradiated by a 10 ps laser pulse with a fluence of 0.45 J/cm^2^.

Figure 11 presents the time evolution of the electron and lattice temperatures as calculated by a two-temperature diffusion model for a Au target that is irradiated by a 10 ps laser pulse with a fluence of 0.45 J/cm^2^. The two systems reach equilibrium at about 70 ps after the laser pulse onset. The maximum lattice temperature that is reached is 3300 K, i.e., higher than the boiling point of Au (3081 K). Note that as the target is heated, temperature gradients of about 10^14^ K/s are being developed.

**Figure 12:**
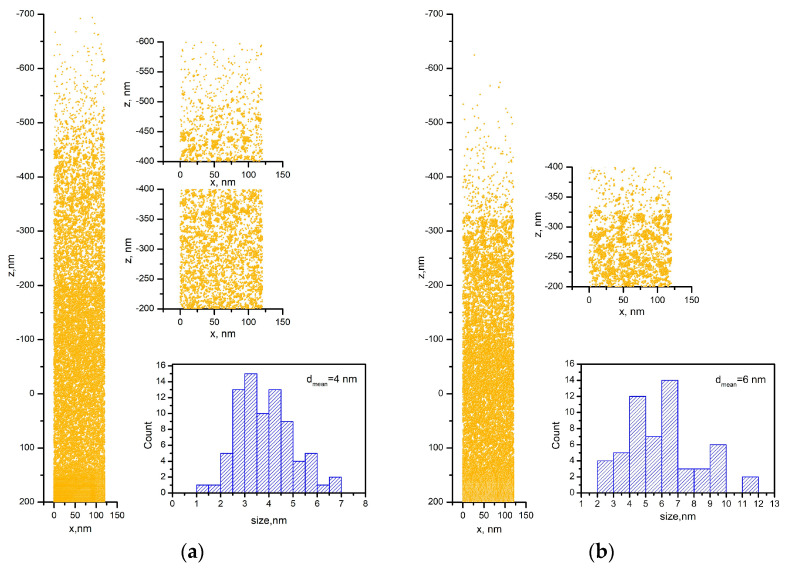

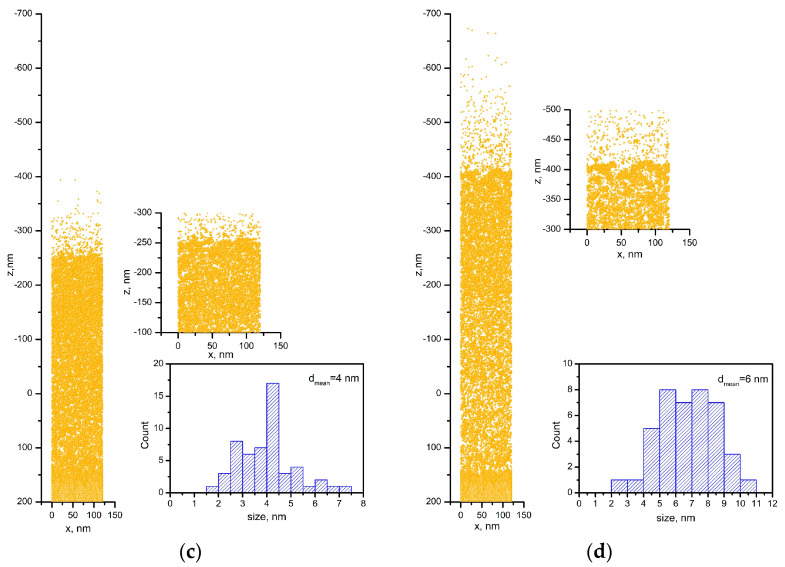
Snapshot of Au target irradiated by a 10 ps laser pulse in vacuum 50 ps after the laser pulse onset with a fluence of (**a**) 0.45 J/cm^2^ and (**b**) 0.25 J/cm^2^. Snapshot of Au target irradiated by a 10 ps laser pulse in air at a fluence of 0.45 J/cm^2^ (**c**) 50 ps and (**d**) 80 ps after the laser pulse onset.

The response of the material irradiated under such conditions is provided in Figure 12. Figure 12a shows a snapshot of the simulated system under the condition 50 ps after the laser pulse onset, when a laser fluence of 0.45 J/cm^2^ is applied to the Au target in vacuum. The strong heating of the material results in a rapid expansion; a detailed view shows that the material starts to decompose into single atoms and clusters. The expanded view presented in the figure shows that the clusters that are obtained have irregular shapes. Their size was determined under the assumption that they are spherically shaped with a diameter equal to the mean value of the size along the *x* and *z* axes. The size distribution of the clusters shows sizes in the range of 1–7 nm (Figure 12a) with a mean diameter of 4 nm.

Figure 12b is a snapshot of the simulated system 50 ps after the laser pulse onset, when the laser fluence that is applied to the Au target in vacuum is 0.25 J/cm^2^. We observe that when a lower fluence is applied to the Au target, its material undergoes similar decomposition. However, the corresponding size distribution demonstrates the formation of bigger clusters, with the mean diameter being estimated as 6 nm (Figure 12b).

Snapshots of Au targets irradiated by a 10 ps laser pulse at a fluence of 0.45 J/cm^2^ in air are presented in Figure 12c,d. The development of the simulated system in air was obtained by limiting the velocities of the species obtained in a vacuum. Figure 12c is a snapshot of the simulated system 50 ps after the laser pulse onset. The decomposition of the irradiated material leads to formation of clusters with sizes ranging from 2 nm to 7 nm with a mean diameter of 4 nm (see size distribution in Figure 12c). Figure 12d displays a simulation of the situation 80 ps after the laser pulse onset; the clusters have grown in size, with a mean diameter reaching 6 nm (see size distribution of Figure 12d). The presence of single atoms in the simulated system in air should be noted.

## 4. Discussion

The interaction of high-power ultrashort laser pulses with metals results in strong heating of the absorbing volume followed by rapid melting of the material. In the case of *fs* pulses, the heating takes place at a nearly constant volume. If the temperature reaches a critical value, the usual relaxation mechanism is phase explosion or homogeneous decomposition of the overheated material into gas and liquid droplets [31]. In the case of picosecond pulses, the material is also rapidly melted, but it may undergo expansion, even during the laser pulse. In such a case, the decomposition mechanism can consist of liquid fragmentation [32], where the fast expansion of the overheated liquid results in the development of density fluctuations within the volume, which further evolve into material decomposition into atoms and liquid droplets.

Based on the above scenario, one can conclude that under the conditions discussed, the decomposition of the material occurs via fragmentation rather than phase explosion, as the maximal temperature that is achieved is significantly lower than the critical temperature for gold (~5000 K) (Figure 11). In the case of metal oxides, other studies [33,34] have demonstrated that the ablation of metal oxides by ultrashort laser pulses also leads to decomposition of the material into nanoparticles. It is also considered that the absorption of the ultrashort laser pulses is realized via multiphoton absorption as the absorbing material is rapidly transformed into a “metallic state” with a high concentration of free electrons. Such effects are difficult to simulate due to a lack of reliable data for the materials at such a state. However, based on the above comments, we can assume that the ablation mechanisms of metal oxides could also be attributed to fragmentation.

To summarize, ablation by *ps* laser pulses leads to the production of nanoparticles regardless of the ambient pressure. As demonstrated by the MD simulation, they originate from an ablation mechanism that leads to a direct decomposition into nanoparticles. However, the morphology of the samples shows a clear difference when the ambient pressure is changed (Figure 2, Figure 4, Figure 7 and Figure 9). It is evident that increasing the pressure from vacuum to atmospheric transforms the thin-film morphology that is normally obtained in vacuum to the highly porous structure formed at atmospheric pressure (Figure 2, Figure 4, Figure 7 and Figure 9). In general, the morphology of the samples deposited in vacuum represents a flat thin film with a significant amount of round-shaped nanoparticles/droplets with sizes depending on the material deposited (Figure 2a, Figure 4a, Figure 7a and Figure 9a). Obviously, the size of these droplets is smaller for the samples prepared by ablation of Pt (compared with Au) and ZnO (compared with TiO_2_) targets. We attribute this difference in the size of the droplets to the different ablation thresholds of the materials. Such droplet formations are typical for the classical PLD process performed by nanosecond (*ns*) laser pulses, as their origin is associated with the ejection of molten material due to the overheating of the target [35].

It should be noted that the size of the droplets formed by *ps*-PLD is smaller than that obtained by *ns*-PLD, which is probably related to the limited heat-affected zone when the target is irradiated by ultrashort laser pulses. However, the temperature that is attained on the target surface is considerably higher than the melting point of the material used in this work, as the calculation for the Au target predicts. This is why one should not be surprised to observe particles/droplets on the surface of the sample that is produced by *ps* ablation. It should also be mentioned that the higher the fluence that is applied on the target, the more droplets that will be produced. In this work, we used a fixed laser fluence for the ablation of all target materials independently of their ablation threshold. Furthermore, the microstructure of the samples shows that these particles are embedded in the sample structure (Figure 3a, Figure 6a and Figure 8a). The response of the Au target under a 10 ps irradiation in vacuum consists of decomposition of the material into single atoms and clusters (Figure 12a). Thus, the “building blocks” of the samples deposited in vacuum are single atoms and clusters/nanoparticles that are directly formed at the stage of material decomposition. This way, a thin film with embedded nanoparticles is grown on the substrate via *ps* target ablation.

It should be noted that the experimental results are in good agreement with the results obtained by simulation (Figure 1a and Figure 12a). Note that Figure 1a presents the size distribution of the smallest nanoparticles produced. In the experiments, the laser beam has a Gaussian shape, which means that the real fluence applied to the target will be in a tiny range as the maximum value is 0.45 J/cm^2^. In the simulations, for simplicity, a constant value for laser fluence was used; therefore, simulations at a lower laser fluence were also implemented (Figure 12b). The results demonstrate that a broader size distribution should be expected due to the Gaussian shape of the laser beam.

Complex 3D nanostructures are formed when the deposition process is performed in air (Figure 2b, Figure 4b, Figure 7b and Figure 9b). Similar structures were previously obtained and reported when *fs* or *ns* laser pulses were used for ablation at atmospheric pressure [36,37,38,39]. These structures are composed of particles with nanometer-scale sizes; the main mechanism of their formation is the fast condensation of the ablated species [35,36,37,38,39]. In air, the response of the Au target under 10 ps of irradiation also leads to decomposition of the material into single atoms and clusters (Figure 12c). The temporal evolution of the simulated system reveals the growing size of the clusters formed (Figure 12d), which is in good agreement with the experimental results (Figure 1b). However, due to the high ambient pressure, the ejected atoms condense and form nanoparticles and/or nanoaggregates. Thus, the “building blocks” of the samples deposited in air are nanoparticles and nanoaggregates (Figure 3b, Figure 6b and Figure 8b).

It is worth noting that, in spite of the samples being deposited in air, no nitrogen inclusions were detected on their surface. However, the Pt nanoparticles were oxidized in air during their formation. When comparing the surface of the metal oxides deposited in vacuum and in air, the former had a higher percentage of components assigned to the oxygen deficiency state and to the presence of adsorbed species. This allows us to conclude that more defects are formed on the metal oxide surface when the samples are deposited in vacuum.

Finally, the method for samples’ fabrication is a type of sputtering process. Furthermore, the experiments that were performed were very simple (at room temperature, in vacuum or air). The replication of the obtained results demonstrates that the *ps*-PLD method has good reproducibility. Moreover, the results obtained in this work are in agreement with the results previously reported on PLD performed by using *fs* pulses [40].

## 5. Conclusions

Laser ablation using *ps* pulses leads to the production of nanoparticles independently of the ambient pressure. Increasing the pressure from vacuum to atmospheric transforms the thin-film morphology normally obtained in vacuum to a highly porous structure that is formed in air at atmospheric pressure. The morphology of the samples deposited in vacuum is represented by a flat thin film with a significant amount of round-shaped nanoparticles/droplets, with the size of these particles depending on the material deposited. The difference in the size of the droplets is related to the difference in the ablation threshold of the materials. Furthermore, the droplets that are formed by *ps*-PLD are smaller than those obtained by the classical *ns*-PLD, which is probably related to the limited heat-affected area on the target that is irradiated by ultrashort laser pulses. When the deposition process is performed in air, complex 3D nanostructures are formed, which are composed of particles with nanometer sizes, with the main mechanism of their formation being the fast condensation of the ablated species. The decomposition mechanism in the case of a 10 ps laser pulse irradiation of the Au target consists of liquid fragmentation, which further evolves into material decomposition into atomic and liquid droplet components.

## Data Availability

Not available.

## Supplementary Materials

The following supporting information can be downloaded at: https://www.mdpi.com/article/10.3390/ma16196364/s1, Figure S1: XPS spectra of the Au samples deposited (a) in vacuum and (b) in air; Figure S2: XRD patterns of material ablated from the ZnO target in air; Figure S3: XPS spectra of the ZnO samples deposited (a) in vacuum and (b) in air; Figure S4. XRD patterns of material ablated from the TiO_2_ target in air.
