# Supplementary material for: Picosecond Pulsed Laser Deposition of Metals and Metal Oxides
Source: Materials (Basel). 2023 Sep 22;16(19):6364. doi: 10.3390/ma16196364 (PMC10573754; doi:10.3390/ma16196364)
Supplement: Supplementary file 1 [file materials-16-06364-s001.zip › materials-2617644-supplementary.pdf]

Supplementary Materials:

Article

Picosecond Pulsed Laser Deposition of Metals and Metal Oxides

Anna Dikovska, Genoveva Atanasova, Tina Dilova, Aleksandra Baeva, Georgi Avdeev, Petar Atanasov and Nikolay Nedyalkov

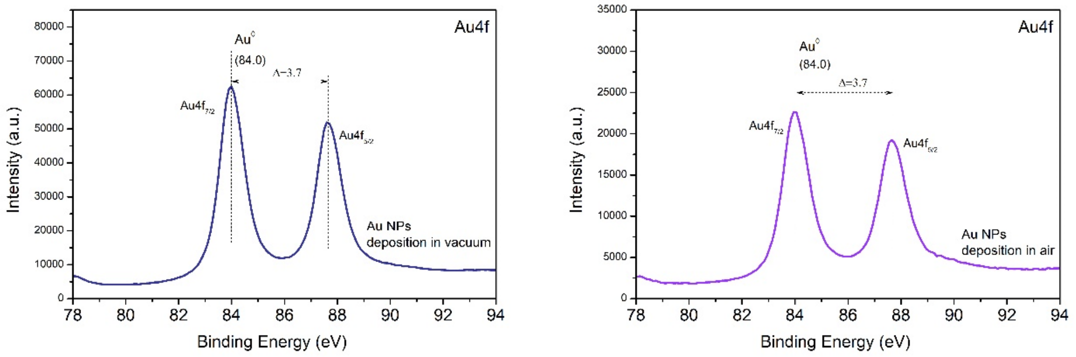

(**a**) (**b**)

Figure S1. XPS spectra of the Au samples deposited **(a)** in vacuum and **(b)** in air.

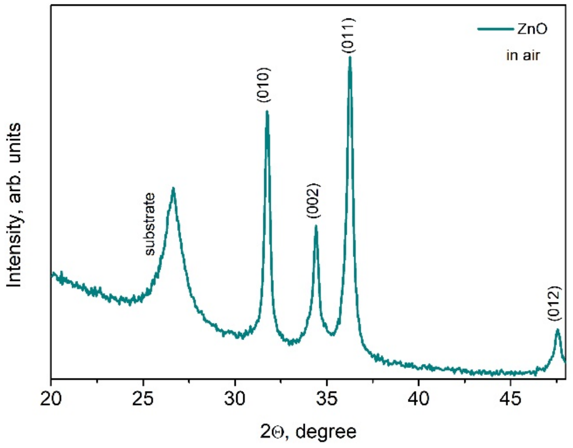

Figure S2. XRD patterns of material ablated from ZnO target in air.

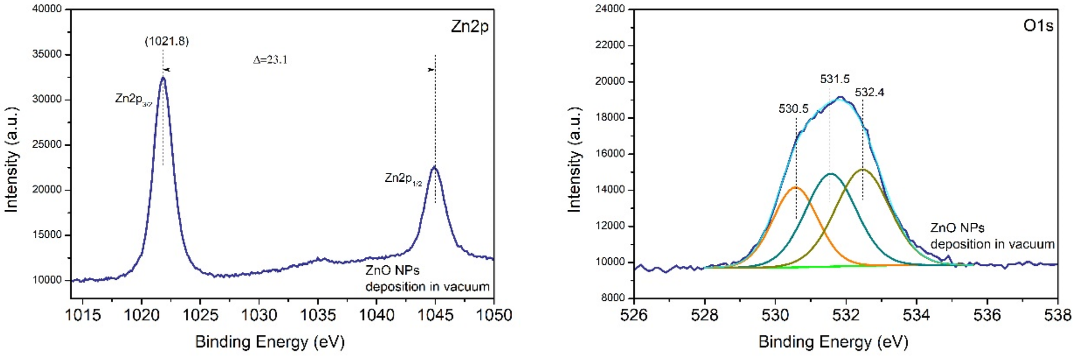

(**a**)

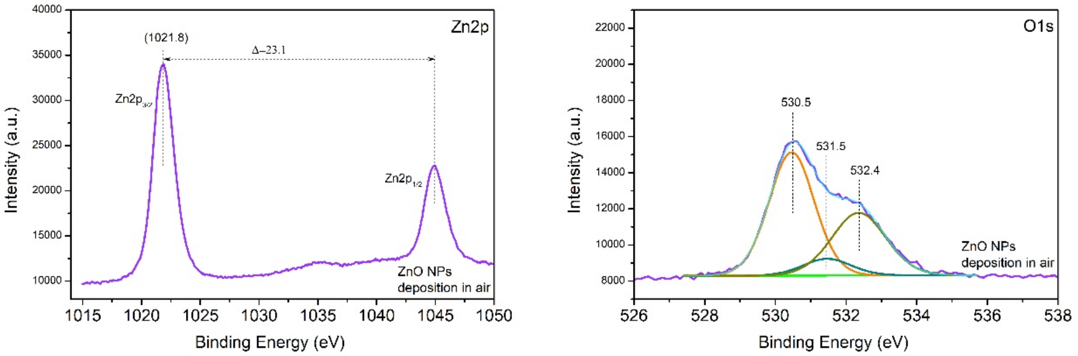

(**b**)

Figure S3. XPS spectra of the ZnO samples deposited **(a)** in vacuum and **(b)** in air.

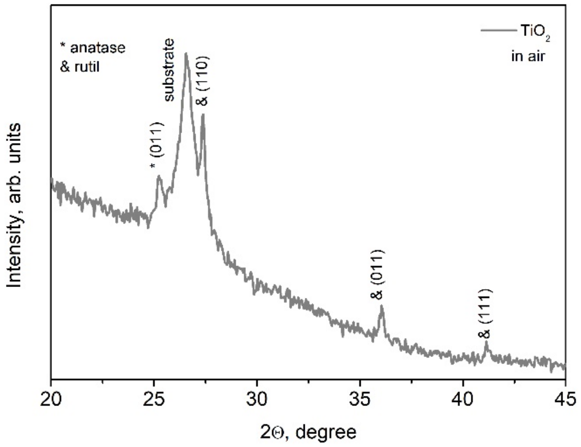

Figure S4. XRD patterns of material ablated from TiO_2 target in air.
